# Odor-induced modification of oscillations and related theta-higher gamma coupling in olfactory bulb neurons of awake and anesthetized rats

**DOI:** 10.3389/fchem.2022.865006

**Published:** 2022-08-01

**Authors:** Ping Zhu, Shuge Liu, Yulan Tian, Yating Chen, Wei Chen, Ping Wang, Liping Du, Chunsheng Wu

**Affiliations:** ^1^ Department of Biophysics, Health Science Center, Institute of Medical Engineering, School of Basic Medical Sciences, Xi’an Jiaotong University, Xi’an, China; ^2^ Key Laboratory of Environment and Genes Related to Diseases, Ministry of Education of China, Xi’an Jiaotong University, Xi’an, China; ^3^ Biosensor National Special Laboratory, Department of Biomedical Engineering, Zhejiang University, Hangzhou, China; ^4^ Key Laboratory for Biomedical Engineering of Ministry of Education, Department of Biomedical Engineering, Zhejiang University, Hangzhou, China

**Keywords:** neuronal oscillations, high gamma, high-frequency oscillations, cross-frequency coupling, odor stimulation

## Abstract

Olfactory gamma oscillations (40–100 Hz) are generated spontaneously in animals and represent the activity of local olfactory bulb (OB) networks, which play important roles in cognitive mechanisms. In addition, high-frequency oscillations (HFO, 130–180 Hz) have attracted widespread attention and are novel neuronal oscillations with a frequency range closer to high gamma oscillations (60–100 Hz, HGOs). Both HGOs and HFOs are distinctly regulated by θ rhythm in the hippocampus. To understand their mediation mechanisms in the OB, we investigated whether local field potential (LFP) oscillations including HGOs and HFOs and even their coupling with theta rhythm are modified by odor stimulation in both freely moving and anesthetized rats. Therefore, we combined electrophysiological technology and cross-frequency coupling analysis approaches to determine the difference in the odor-modulated LFP oscillations between awake and anesthetized rats. The obtained results indicate that LFP oscillations including HGOs and HFOs were differently modified by odor stimulation in animals of both states. However, θ-HGO and θ-HFO coupling were modified in only awake animals. It is suggested that these oscillations and their interactions with theta oscillations may play crucial roles in olfactory network activity. This could pave the way for further understanding the underlying mechanisms of oscillations in OB neurons towards odor sensation.

## Introduction

Neuronal oscillations are ubiquitous in various brain networks, including the thalamus, hippocampus, neocortex, and olfactory bulb (OB), which provide a temporal structure for brain networks communication and processing (Zhang et al., 2018; Urbain et al., 2019; Naggar et al., 2020; Średniawa et al., 2021). This regular electrical activity in neurons is described as several different oscillation bands, including respiration-related δ oscillations (1‒4 Hz), θ rhythm (4–12 Hz), faster oscillations in *β* (15–30 Hz), and *γ* (40–100 Hz) frequency ranges. Β-frequency activity is related to odor sampling and discrimination (Martin and Ravel, 2014). Gamma oscillations are the important symbols of neural network function in the sensory system and play crucial roles in cognitive functions (Vinck et al., 2013).

Gamma oscillations in the OB are dependent on dendritic synaptic interactions between the main output neurons (mitral and tufted cells, M/T cells) and GABAergic interneurons (granule cells) (Osinski and Kay, 2016), which are not only generated spontaneously (Manabe and Mori, 2013) but also modified by odor stimulation (Frederick et al., 2016). Gamma oscillations composed of distinct subbands generated in awake animals exhibit a unique characteristic (Kay, 2003). Low *γ* (LG, 40–60 Hz) and high *γ* oscillations (HGOs, 60–100 Hz) represent different patterns of network interaction. It is considered that paired remote mitral cells are specially synchronized in the LG band. However, HGOs are more restricted in space, representing the local network activity (Lepousez and Lledo, 2013). In addition, scientists described a novel cortical oscillating activity (HFOs) in a frequency range of 130–180 Hz (Scheffer-Teixeira et al., 2012), which was also termed fast *γ* oscillations (Jackson et al., 2011; Tort et al., 2013). In addition to the hippocampus (Jackson et al., 2011; Scheffer-Teixeira et al., 2012) and neocortex (Scheffzük et al., 2011), it is reported that the OB of rodents can generate fast rhythms and is also a source of HFO (Chery et al., 2013; Hunt et al., 2019). Neuron oscillations in different frequency bands represent distinct physiological implications and can interact with each other. The interaction of rhythms in different frequency bands is generally called cross-frequency coupling (CFC). The most famous example of CFC is in the hippocampus, where the amplitude of the *γ* oscillation is modulated by the θ rhythm (Li et al., 2019; Nakazono et al., 2019; Salimi et al., 2021). Recent studies have shown that this θ-*γ* coupling reflects the general mechanism of dynamic information processing in the brain and the strength of coupling changes during learning, long-term, and working memory processes (van Wingerden et al., 2014; Griffiths et al., 2019; Jones et al., 2020; Vivekananda et al., 2021). In addition, there has been evidence that the θ-*γ* coupling is involved in the sensory processing of the cortex, such as olfactory, auditory, and vision (Shusterman et al., 2011; Hamm et al., 2012; Murphy et al., 2020). The OB is an ideal site to investigate whether the phase amplitude coupling (PAC) is involved in sensory processing because of its inherent oscillatory activity associated with odor signal processing. The oscillation activity in the OB can powerfully drive the coherent rhythmic activity in the remote brain region (Ito et al., 2014). In addition, the θ-*γ* coupling in the OB changed during the process of awake immobile and exploring (Zhong et al., 2017).

According to previous studies in extracellular recording or imaging of the OB in anesthetized and awake animals, odor responses of mitral and tufted cells were significantly different (Buzsáki and Watson, 2012). The changes in the OB neuron activity in awake animals are related to the olfactory stimulation, which conveys information about odor recognition and odor value. Therefore, it is crucial to study the odor response of neurons in awake animals. Among many studies involving local field potential information in awake animals, a large number of studies have focused on the critical role of neuronal *γ* oscillations in olfactory perception and cognition, as well as olfactory-related learning and memory (Rosero and Aylwin, 2011; Manabe and Mori, 2013). Scientists found that the PAC between the θ phase and the high *γ* amplitude in the OB of awake rats varied during the learning process (Losacco et al., 2020). Therefore, this modulation plays a role in the early sensory processing of olfaction during the learning phase. This work highlights the important original information about the influences of learning on the *γ* oscillations of OB, providing new insight into the mechanisms of OB information processing. These discoveries about *γ* oscillations also led us to investigate the influences of odor stimulation on the HFO of OB in awake rats. Several studies on the local field potential (LFP) in the OB of anesthetized animals have discussed the mechanism of HFO generation and occurrence in mammals (Średniawa et al., 2021). Although studies on anesthetized animals provide much information, it is crucial to study the neuronal responses of awake animals. We examined the changes in the oscillatory activity of the OB neurons and their coupling activity in rats under two states and how these changes are related to odor stimulation.

Here, we implanted multi-channel micro-electrodes into the OBs of rats, which were utilized to record the LFP signal of M/T cells in the OB of awake and anesthetized rats. We examined the changes in HGOs and HFOs and their coupling with θ oscillations during odor stimulation. It is indicated that the neural oscillations in different frequency bands and their interactions are modified by odor stimulation. Moreover, the differences in odor-induced LFP and HFOs activity between anesthetized and awake animals are clear. In addition, the differences in HGOs and HFOs and even their coupling with θ rhythm during odor stimulation is also obvious. These findings provide new clues for understanding the mechanism of neuronal oscillations participating in the communication of the OB network.

## Experiment

### Materials and instruments

We used three adult male Sprague–Dawley rats (250–300 g) purchased from the experimental animal center of Xi’an Jiaotong University. An 8-channel microelectrode array was handmade with a 38-μm diameter formvar-coated nichrome microwire electrode (AM system, WA; #761500). The signal recording system used was a wireless W2100-system (Multichannel Systems, Germany), a data acquisition system consists of a receiver and interface board. The system can be connected to a computer *via* a USB. The odorants used in the experiment include nonanoic acid (CH_3_(CH_2_)_7_COOH, NA, Tokyo Chemical Industry, P0952) and amyl hexanoate (C_11_H_22_O_2_, AH, Macklin, P859158, China). Dental cement was purchased from luxatemp (Dental Milestones Guaranteed, America). A brain stereotaxic instrument was used (RWD Life Science, China) and the fixative was paraformaldehyde (4%, PI105, ZHONGHUIHECAI, China).

### Surgery, odor stimulation, and signal recording

The microwire electrode array was implanted into the OB of rats. After being anesthetized with chloral hydrate (intraperitoneal injection, 4 ml/kg), the rats were individually fixed on a brain stereotaxic instrument. A craniotomy was performed to expose the OB and related brain areas (Supplementary Figure S1A). The microelectrode array was implanted into the mitral/tufted (M/T) cell layer of OB according to specific coordinates (Anterior-Position: +8.2 mm, Medial-Lateral: −1.0 mm, and Doral-Ventral: +0.8 mm) with the real-time synchronous recording of signals (Supplementary Figure S1B). The depth of the electrode tip was determined by monitoring the configuration of neuron activity of OB (Supplementary Figures S1C,D). After implantation, the microelectrode array was fixed onto the head with the dental cement. Then, the headstage was disconnected from the microelectrode array. The use of animals was approved by the Medical Ethics Committee of Xi’an Jiaotong University.

We performed the electrophysiological LFP signal recording after the rats recovered from the surgery (3‒5 days). Before the odorant stimulation, we connected the microelectrode array with the headstage of a multichannel system with a sampling rate of 25 kHz. The odorant was diluted in the mineral oil to 10^−3^ M. Then, the odor was delivered to the nose of the awake rat through a glass dish containing an odor-saturating filter paper. Each stimulation lasted 2 s. Nonanoic acid is a colorless, oily liquid with a specific smell. It is naturally found in bananas, beer, and bread. Amyl hexanoate has a sweet, fragrant smell, naturally found in apples, grapes, and other fruits. Obvious sniffing behavior could be observed during the odor stimulus. This could be used to determine the duration of the odor sample. The cases in which the animal did not have a reliable odor-related behavioral response after the odor presentation were not included in the trial. A 5 min refreshing was performed between each odor stimulation to refresh the room. Three rats were used in this study. Two kinds of odor stimulation were applied to each rat under anesthesia and awake state. Each rat received four odor stimuli (nonanoic acid and amyl hexanoate twice each) under each state (anesthesia and awake). Three rats received 12 trials of odor stimulation in total in each state.

After being anesthetized, the rats were perfused through the left ventricle with 400 ml of paraformaldehyde. The bilateral OB was removed and post-fixed with paraformaldehyde to inspect the recording region.

### Data analysis

Filter and spectral analysis: The recorded electrophysiological data were analyzed offline using the software of MATLAB (Mathworks, Inc.) and the software program Spike2 (Cambridge Electronic Design). LFPs were obtained by filtering the original signals using a 2nd-odor Butterworth band-pass filter at 0.1–200 Hz, which were then divided into different frequency bands: θ oscillations (4–12 Hz), high *γ* oscillations (60–100 Hz), and high-frequency oscillations (130–180 Hz). The wavelet transform of the signal was realized by a MATLAB function ‘cwt.’ The fast Fourier transform was used to calculate the power spectra of oscillations for the 0–1 s period after the stimulus onset and compared with the 1 s pre-stimulus baseline. The Data were analyzed using the repeated-measures ANOVA followed by the Fisher’s least significant difference (LSD) post hoc test, with the statistical difference determined at *p* < 0.05. Graphpad Prism (Graphpad Software Inc., United States) was used for the statistical analysis.

For the cross-frequency coupling (CFC), the modulation index (MI) was used to measure the intensity of phase-amplitude coupling between the two frequency ranges of interest: The phase modulating (f_P_) and amplitude modulating (f_A_) frequency bands. The computation of the MI was as described elsewhere (Tort et al., 2010). Briefly, the steps required are as follows: The raw signal was filtered at the two frequency ranges (f_P_ and f_A_) (Supplementary Figures S2A–C). The standard Hilbert transform was applied to the raw signal to obtain the time series of the phase of f_P_ and the amplitude envelope of f_A_ (Supplementary Figures S2D,E). The phase of f_P_ was then binned into eighteen 20° intervals, and the mean amplitude of f_A_ over each phase bin was calculated. Next, the mean amplitude of f_A_ in each bin was normalized so that the sum of all bins equaled to 1 giving rise to a phase-amplitude distribution (Supplementary Figure S2F). A uniform distribution of phase-amplitude indicated no phase-amplitude coupling between the two frequency ranges of interest. Therefore, MI estimating the KL distance is defined as the measure that quantifies the deviation of the phase-amplitude distribution from the uniform distribution (Tort et al., 2010). The stronger the phase-to-amplitude modulation, the larger the MI value (Tort et al., 2008). The comodulogram was obtained by calculating the MI of multiple band pairs and displaying the results on the two-dimensional pseudo-color map (Supplementary Figure S2G). The bandwidths of phase-frequency and amplitude-frequency were 4 and 10 Hz, respectively. The coordinate of each MI was its center frequency. Here, the phase modulating frequency (θ oscillation) is denoted as one dimension in the comodulogram. Amplitude modulating frequency (*γ* oscillation) is denoted as another dimension. The color in the comodulogram refers to the strength of the modulation.

## Results

### Odor responses in anesthetized and awake rats

The LFP oscillation of the OB was modulated by odor stimulation. This modulation of LFP oscillations in the OB by odor has been widely described in anesthetized and awake animals (Cenier et al., 2008; Aylwin et al., 2009; Kum et al., 2019). In the olfactory system of awake mammals, particularly when animals freely explore new environments without any olfactory stimulation, the OB exhibits regular *γ* oscillations, which has been widely studied (Rojas-Líbano and Kay, 2008). We compared how the LFP frequency band oscillations of the OB neurons in the anesthetized and awake rats were modulated by odors. In our study, multichannel microelectrodes were implanted into the OB in the anesthetized and awake rats. These electrodes were then used to record olfactory neuron oscillations in surgically recovered rats. The detected oscillations were explored in the time and frequency domains. In the time-domain index, we focused on the included signal waveform and amplitude of the oscillating frequency band. While in the frequency domain, the power spectrum and spectrum diagram were calculated to analyze the maximum power and time-frequency information in the pseudo-color mode, respectively. Pseudo-color window functions can be used to display the energy density spectrum of each short period so that time-frequency analysis can be carried out visually.

**FIGURE 1:**
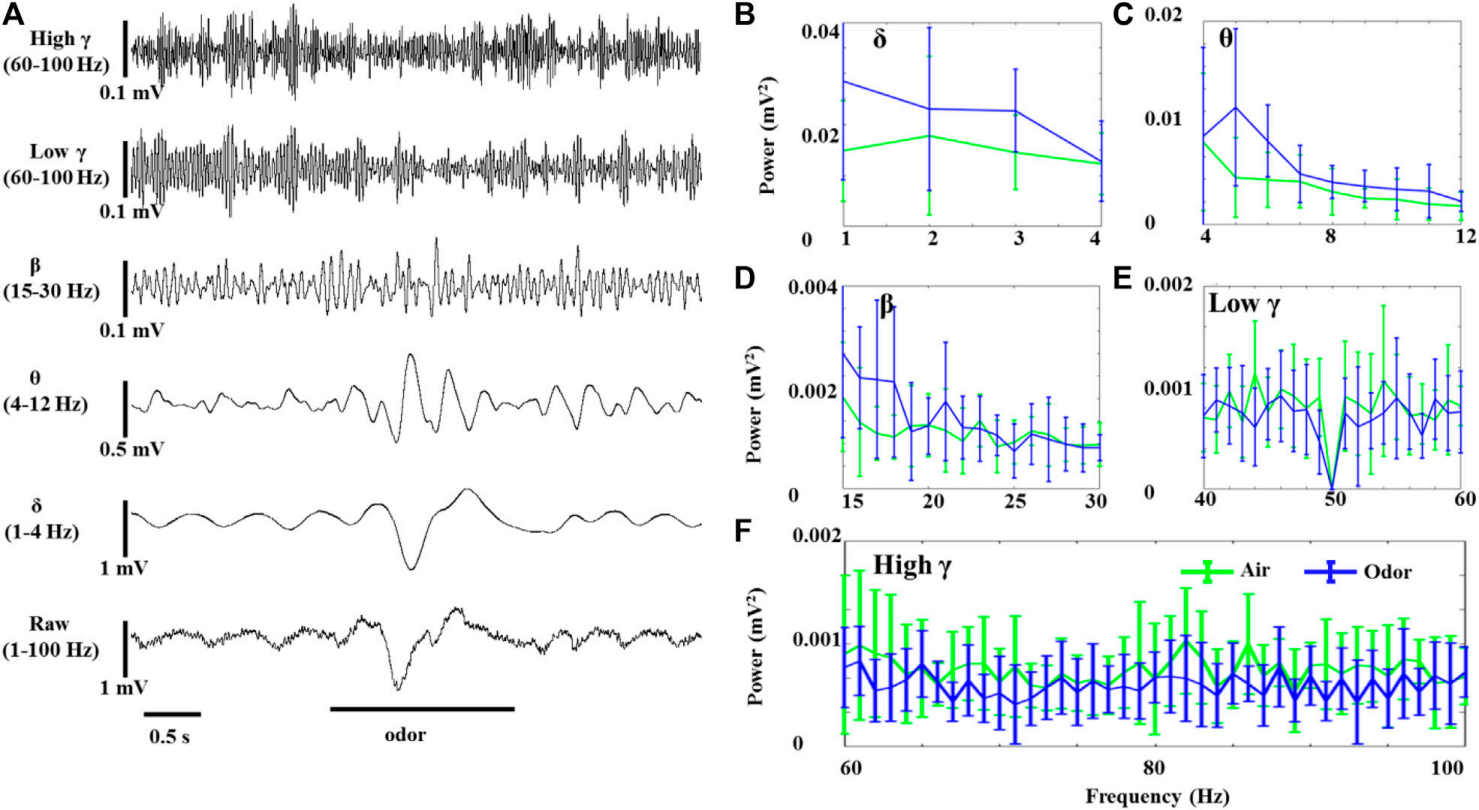
Odor-evoked LFP (1–100 Hz) responses in the OB of awake rats. **(A)** Typical OB LFP traces recorded under nonanoic acid (NA) stimulation. Shown are the band-pass filtered traces of δ (1‒4 Hz), θ (4–12 Hz), *β* (15–30 Hz), low *γ* (40–60 Hz), and high *γ* (60–100 Hz) activity, as well as full band LFP (1–100 Hz). The mean fast Fourier transform (FFT) power spectrum shows the distribution of energy from the δ- **(B)**, θ- **(C)**, *β*- **(D)**, low *γ*- **(E)**, and high *γ*-bands **(F)** across three rats under air (green line) and nonanoic acid (NA) (blue line) stimulation. The trial-averaged data are presented as the mean ± SEM, *n* = 12, 4 trials per rat.

LFP odor responses of three rats were recorded under anesthesia and awake conditions. The spontaneous activity of the OB neurons was composed of slow oscillation with high amplitude and regular fluctuation and fast wave with low amplitude and irregular fluctuation. Rhythmicity changes were observed in all the recorded animals in significant ways in response to odor stimuli. Figure 1 shows the effects of odor stimulation on the *δ*-, θ-, *β*-, LG-, and HG-bands’ powers recorded in the OB of awake rats (Figure 1A). In the OB of awake animals, odor exposure caused significant δ-, θ-, and *β*-oscillations, accompanied by rapid responses. The δ-, θ-, and *β*-bands powers increased during odor stimulation and gradually returned to baseline after odor stimulation (Figures 1B–D). However, the OB’s low *γ*- and high *γ*-bands’ powers in the awake rats decreased under odor stimulation (Figures 1E,F). As reported in the previous articles that studied *γ* oscillations in the olfactory system, odor stimulation modifying HGOs does not mean that *γ* oscillations increase induced by odor but significantly decrease (Ravel et al., 2003; Liu et al., 2020).

**FIGURE 2:**
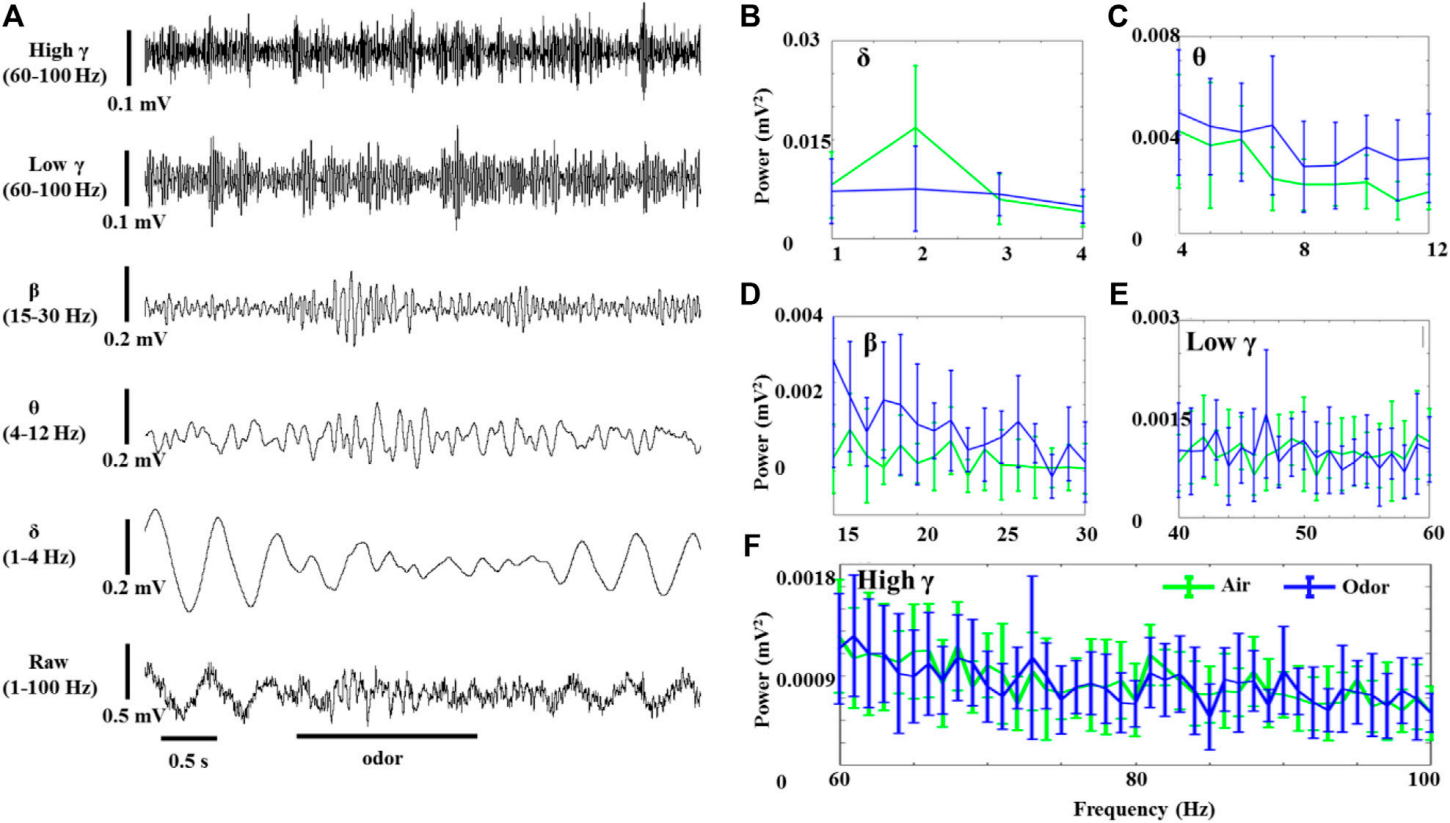
Odor-evoked LFP (1–100 Hz) responses in the OB of anesthetized rats. **(A)** Typical OB LFP traces recorded under nonanoic acid (NA) stimulation. Shown are the band-pass filtered traces of δ (1‒4 Hz), *β* (15–30 Hz), low *γ* (40–60 Hz), and high *γ* (60–100 Hz) activity, as well as full band LFP (1–100 Hz). The Mean fast Fourier transform (FFT) power spectrum showing the distribution of energy from the δ- **(B)**, θ- **(C)**, *β*- **(D)**, low *γ*- **(E)**, and high *γ*-bands **(F)** across three rats under air (green line) and NA (blue line) stimulation. The trial-averaged data are presented as the mean ± SEM, *n* = 12, 4 trials per rat.

The OB neurons of anesthetized rats also showed significant LFP oscillations without odor stimulation. Figure 2 shows the effects of odor stimulation on the δ-, θ-, *β*-, low *γ*-, and high *γ*-powers recorded in the OB of anesthetized rats (Figure 2A). In contrast to awake rats, odor stimulation significantly reduced the δ oscillations of the OB neurons in the anesthetized rats (Figure 2B). However, the increase of θ and *β* power in the odor-induced anesthetized rats was similar to that in the awake rats (Figures 2C,D). The *γ* power (Including low *γ* and high *γ*) of the OB in the anesthetized rats did not change significantly before and after odor stimulation.

**FIGURE 3:**
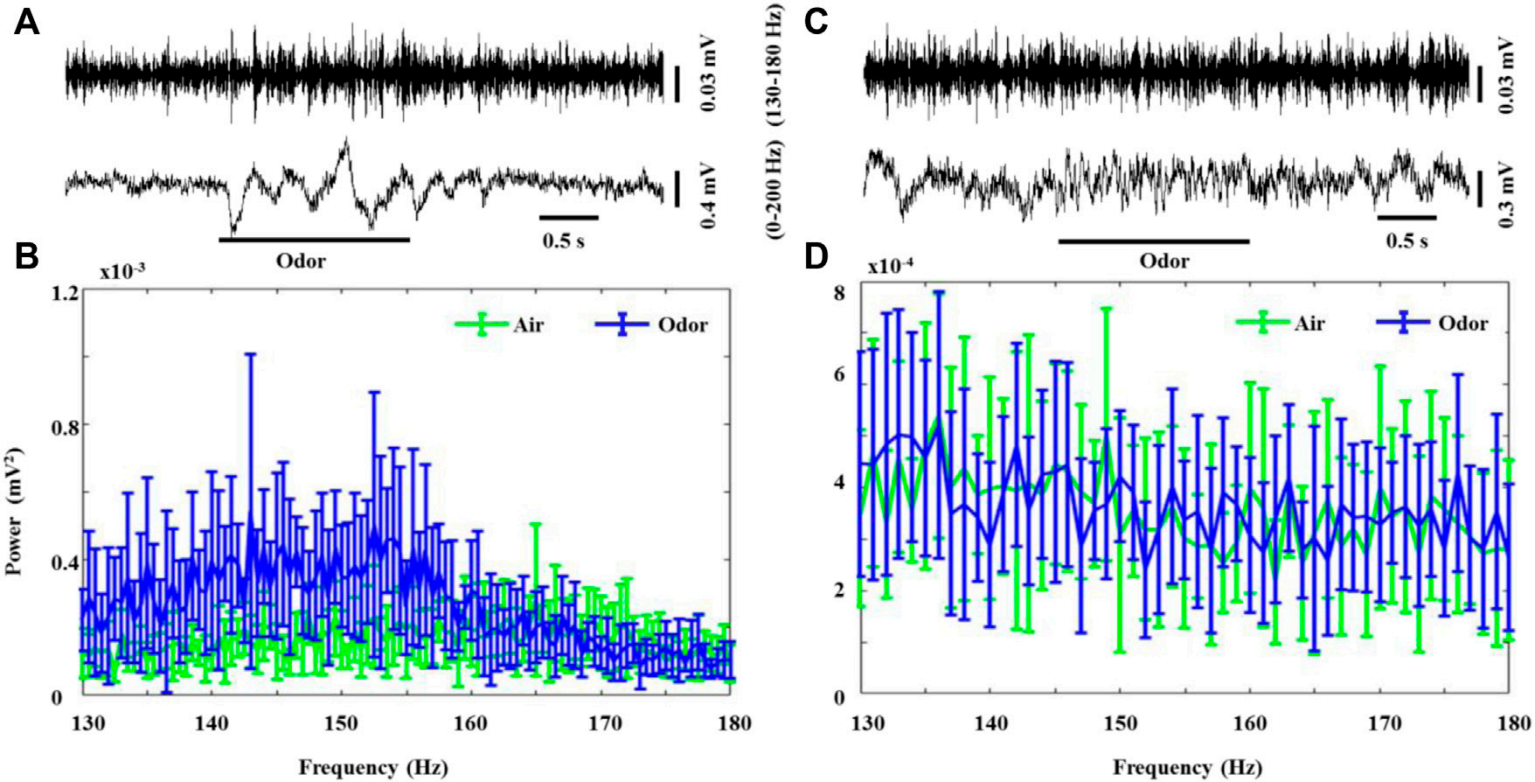
Odor-evoked high-frequency oscillations (130–180 Hz) responses in the OB of awake and anesthetized rats. **(A)** Typical OB 0–200 Hz activity and HFO traces from an awake rat under nonanoic acid (NA) stimulation. Shown are the HFO activity and 0–200 Hz activity. The mean fast Fourier transform (FFT) power spectrum showing the distribution of energy from HFO-bands **(B)** across three awake rats under air (green line) and NA (blue line) stimulation. The trial-averaged data are presented as the mean ± SEM, *n* = 12, 4 trials per rat. **(C)** Typical OB 0–200 Hz activity and HFO traces from an anesthetized rat under nonanoic acid (NA) stimulation. Shown are the HFO activity and 0–200 Hz activity. The mean fast Fourier transform (FFT) power spectrum showing the distribution of energy from HFO-bands **(B)** across the three anesthetized rats under air (green line) and NA (blue line) stimulation. The trial-averaged data are presented as the mean ± SEM, *n* = 12, 4 trials per rat. The short black line represents the duration of 0.5s, and the long black line represents the duration of the odor stimulation.

Another pattern of odorant-induced olfactory oscillations was found in some recorded neurons, which erupted in weak but steady bursts of rapid LFP oscillations (130–180 Hz, HFO) in the absence of odor stimulation in both the awake and anesthetized rats. Interestingly, odor stimulation led to a significant increase in the HFO power in awake rats (Figure 3A, B). After odor stimulation, the recorded neurons recovered their spontaneous weak HFO. The anesthetized rats also showed significant HFO oscillations, but odorant stimuli did not modulate these high-frequency oscillations (Figure 3C, D). To the best of our knowledge, our study is the first to describe that HFOs produced by the OB neurons in awake rats are modified by odor stimulation, providing new insights into the role of OB neuron HFOs in olfactory processing. There is some evidence that tufted cells, rather than mitral cells, generate outputs in this frequency range (Nagayama et al., 2004; Manabe and Mori, 2013). Previous studies introduced high-frequency neural population oscillations generated by the OB of rats under ketamine anesthesia (Hunt et al., 2019). Our study describes for the first time that HFOs generated by the OB neurons of awake rats are modified by odor stimulation, which provides a new understanding of the role of HFOs in the OB neurons in olfactory processing.

**FIGURE 4:**
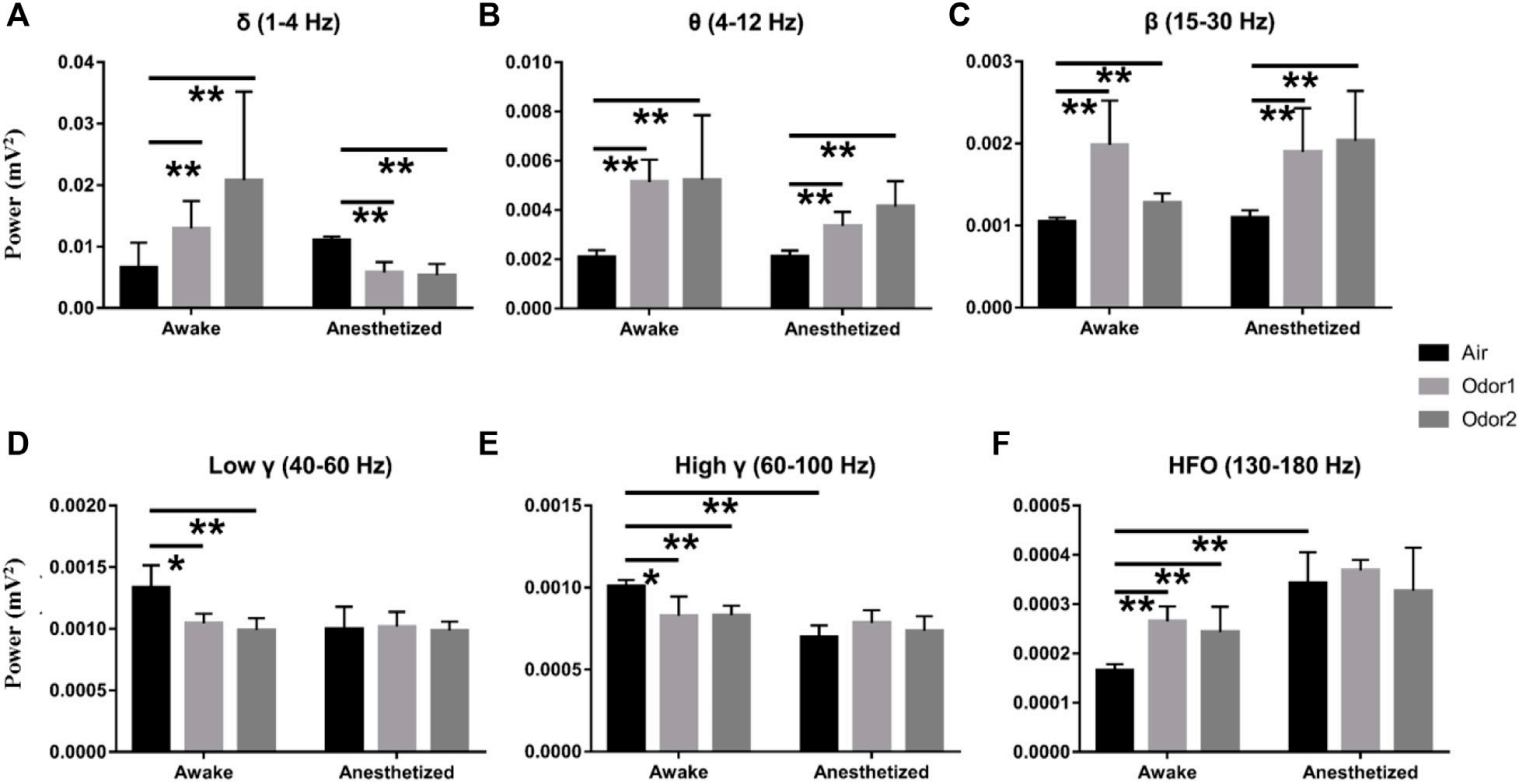
Histogram displaying the quantification of power spectrum in the δ- **(A)**, θ- **(B)**, *β*- **(C)**, low *γ*- **(D)**, high *γ*- **(E)**, and HFO-bands **(F)** in 3 awake and 3 anesthetized rats under the stimulation of air, nonanoic acid (NA, odor 1), and amyl hexanoate (AH, odor 2) with 2 trials per odor per rat. **p < 0.01, *p < 0.05,and ANOVA followed by Fisher’s least significant difference (LSD).

To more accurately examine the difference of odor-modulated LFP activity of the OB neurons between the anesthetized and awake rats, the LFP power was quantitatively analyzed. For the δ power, the increase of odor-induced power in the awake rats was significant, and the decrease of odor -induced power in the anesthetized rats was also significant (Figure 4A). A quantitative comparison of the mean power before and during odor stimulation showed that odor stimulation caused a significant increase in θ and *β* powers in both the anesthetized and awake rats (Figures 4B,C). At the same time, odor stimulation also caused a significant decrease in the *γ* power (low *γ* and high *γ*) of the OB in the awake rats (Figures 4D,E). The enhancement of HFO power of OB in the awake rats induced by odor stimulation was also significant (Figure 4F). In addition, some interesting results were obtained in the quantitative analysis of LFP power. First, there was no significant difference in the LFP responses of different frequency bands in the OB neurons between the conscious and anesthetized rats induced by the two odors used in the study. The decrease in LFP power caused by the odor sampling did not depend on the chemical nature of stimuli but on the behavioral significance. As reported by Frederick et al. (2016), the modulation of *γ* power showed differences in different olfactory tasks. Second, in the absence of odor stimulation, the OB neurons of awake rats exhibited stronger HGO and weaker HFO than anesthetized rats, which is consistent with that reported in previous studies (Manabe and Mori, 2013; Średniawa et al., 2021).

### Modifying θ-HGO/HFO coupling during odors

Neural oscillations in distinct frequency bands play different roles in the brain network communication and computation, and interacting oscillations help to regulate this integration of brain activity (Mofleh and Kocsis, 2021). Interactions between different frequency bands termed CFC, particularly the phase-amplitude CFC, have been reported in several brain regions, highlighting their importance in learning and memory. We first used multichannel microelectrodes to record LFPs from the three freely moving rats with odor presentation to characterize the phase-amplitude CFC in the OB during the odorant presentation. Then, we analyzed the CFC between the θ phase and the amplitude of the two *γ* subbands in the LFP recorded in the OB by referring to the previously reported method (Tort et al., 2010).

**FIGURE 5:**
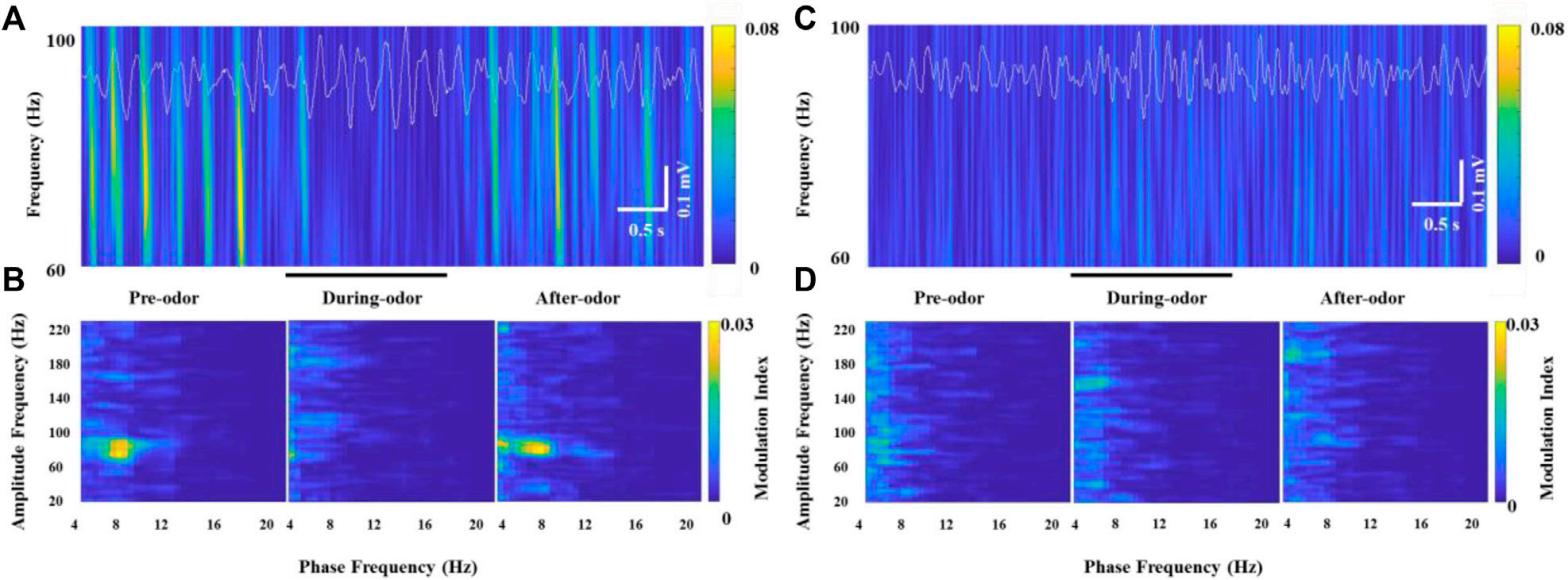
Example of specific coupling between θ and high *γ* oscillations (60–100 Hz) in an awake rat **(A,B)** and an anesthetized rat **(C,D)** during odor stimulation. **(A)** Wavelet spectrograms and superimposed θ signals (4–12 Hz, white traces) from the recorded OB neurons in an awake rat pre-odor (left), during odor (middle), and after odor (right). **(B)** Comodulation maps obtained from the OB neurons of a freely moving rat pre-odor (left), during odor (middle), and after odor (right). **(C)** Wavelet spectrograms and superimposed θ signals (4–12 Hz, white traces) from the recorded OB neurons in an anesthetized rat pre-odor (left), during odor (middle), and after odor (right). **(D)** Comodulation maps obtained from the OB neurons of an anesthetized rat pre-odor (left), during odor (middle), and after odor (right).

In all the awake freely moving rats, we found prominent coupling between θ and *γ* oscillations in the OB. Scheffer-Teixeira et al. (2012) observed the θ-high *γ* coupling and θ-high frequency oscillation coupling with different simultaneously recorded electrodes in the CA1 region of the hippocampus. Similarly, we observed the θ phase modulation of two non-overlapping frequency oscillations, HGO or HFO, in simultaneously recorded electrodes implanted into the OB. More specifically, the HGO amplitude was visibly phase-locked to the phase of the θ rhythm according to wavelet spectrograms and θ traces in recorded HG burst neurons (Figure 5A). We confirmed the phase-amplitude coupling between θ and HGO by cross-frequency comodulation analysis and power spectrum of θ in awake rats (Figure 5B, left panel). However, along with the decrease of the *γ* power during odor stimulation, this phase-amplitude coupling was also significantly weakened or even disappeared (Figure 5B, middle panel), which resumed as before after the odor stimulus was completed (Figure 5B, right panel). It is worth noting that during each repeated trial (2 min, in which the odor stimulation lasted for 2 s), the rats were freely moving, during which the OB exhibited a strong θ-*γ* coupling. Unlike the transition of phase-amplitude coupling in different behavioral states (Zhong et al., 2017), the decrease of the *γ* power during odor stimulation led to a significant decrease in the modulation of the θ phase to the *γ* amplitude. However, we did not find this cross-frequency coupling in the OB of anesthetized rats, nor was it modulated by odor stimulation (Figures 5C,D).

**FIGURE 6:**
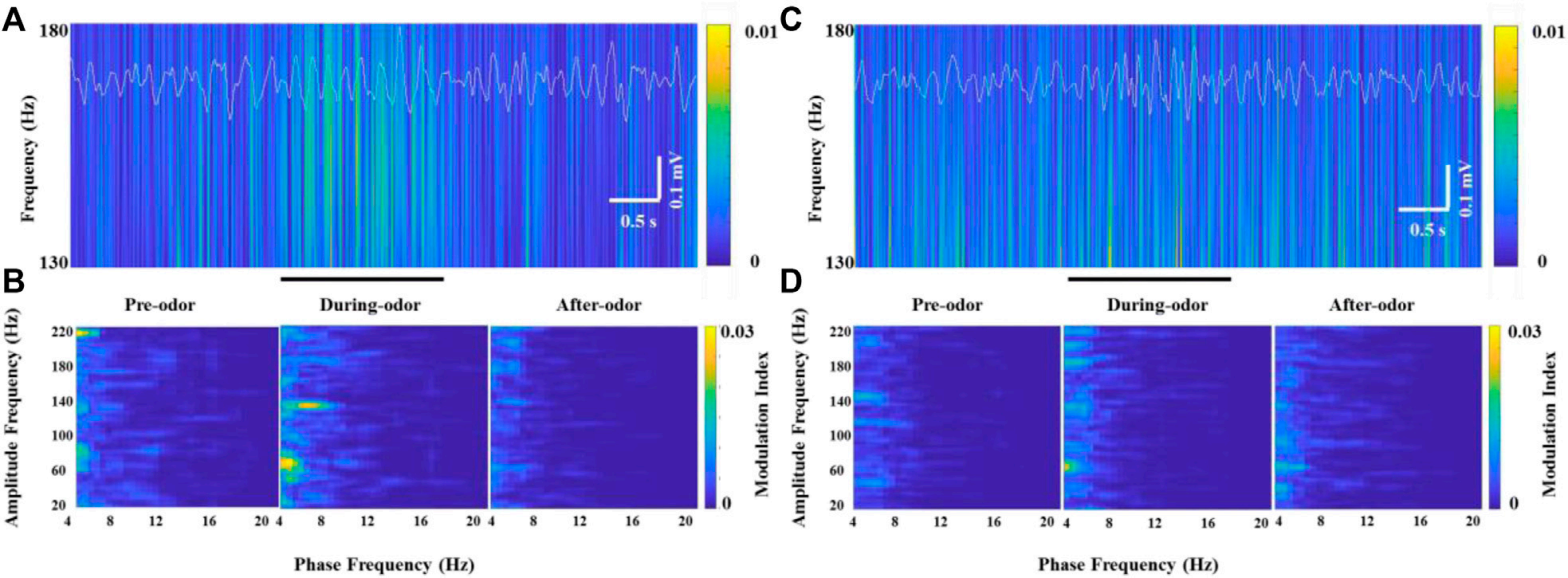
Example of specific coupling between θ and high-frequency oscillations (130–180 Hz) in an awake rat **(A,B)** and an anesthetized rat **(C,D)** during odor stimulation. **(A)** Wavelet spectrograms and superimposed θ signals (4–12 Hz, white traces) from the recorded OB neurons in an awake rat pre-odor (left), during odor (middle), and after odor (right). **(B)** Comodulation maps obtained from the OB neurons of a freely moving rat pre-odor (left), during odor (middle), and after odor (right). **(C)** Wavelet spectrograms and superimposed θ signals (4–12 Hz, white traces) from the recorded OB neurons in an anesthetized rat pre-odor (left), during odor (middle), and after odor (right). **(D)** Comodulation maps obtained from the OB neurons of an anesthetized rat pre-odor (left), during odor (middle), and after odor (right).

Unlike HGO, we did not find a prominent θ-HFO coupling activity in the neurons that generated significant HFOs in the OBs of freely moving rats without odor stimulation when the neurons burst weak but stable HFOs (Figure 6A and Figure 6B, left panel). Along with the significantly increasing HFO power and amplitude, prominent modulation of HFO activity by the θ phase in the OB was observed during odor stimulation (Figure 6B, middle panel). Similarly, the amplitude and power of HFO and CFC resumed as before and after odor stimulation (Figure 6B, right panel). This odor-modulated cross-frequency coupling was not observed in anesthetized rats (Figures 6C,D).

**FIGURE 7:**
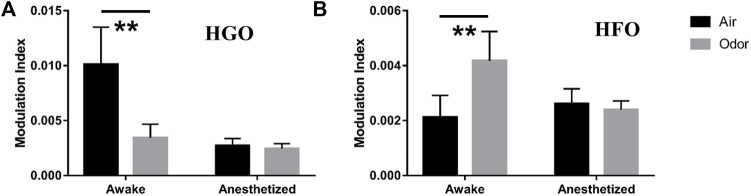
**(A)** Mean modulation index values of Θ-HGO coupling (pair of frequencies ranging 4–12 Hz for phase frequency, 60–100 Hz for Amplitude Frequency) in three awake and three anesthetized rats under air and odors stimulation. **(B)** Mean modulation index values of Θ-HFO coupling (pair of frequencies ranging from 4–12 Hz for phase frequency, 130–180 Hz for Amplitude Frequency) in 3 awake and 3 anesthetized rats under air and odors stimulation. The trial-averaged data are presented as the mean ± SEM, *n* = 12, 4 trials per rat. **p < 0.01, *p < 0.05, and ANOVA followed by Fisher’s least significant difference (LSD).

To quantify the θ-HGO coupling and θ-HFO coupling in the OB, we calculated the mean value of the modulation index of multiple frequency pairs in the two sets of frequency bands. For the θ-HGO coupling, we calculated the mean modulation index of 12 repeated trials of 369 pairs of frequencies from 2‒6 Hz (central frequency at 4 Hz) and 55–65 Hz (central frequency at 60 Hz) to 10–14 Hz (central frequency at 12 Hz) and 95–105 Hz (central frequency at 100 Hz) (Figure 7A). For the θ-HFO coupling, we calculated the mean modulation index of 12 repeated trials of 459 pairs of frequencies from 2‒6 Hz (central frequency at 4 Hz) and 125–135 Hz (central frequency at 130 Hz) to 10–14 Hz (central frequency at 12 Hz) and 175–185 Hz (central frequency at 180 Hz) (Figure 7B). The results showed that the average modulation index of θ-HGO coupling in the OB of awake rats decreased significantly under odor stimulation. In contrast, the modulation index of θ-HFO coupling increased significantly. Thus, the θ-HGO coupling and θ-HFO coupling activity in the OB of awake rats were both modulated significantly by odor stimulation. A significant θ-HFO coupling was observed in the OB, the prefrontal cortex (PFC), and ventral striatum (VS.) after injection of ketamine (Hunt et al., 2019), indicating the association between olfactory networks and oscillatory activity in the limbic areas. A cross-frequency coupling between the olfactory and hippocampus in a new context provides evidence of the hippocampus participating in the OB sensory processing (Pena et al., 2017). The finding that odor stimulation enhanced the θ-HFO coupling in the OB of freely moving rats suggests that odor stimulation may modify the communication between the olfactory system and the brain network through oscillation activity.

## Discussion

In this study, we recorded the LFP of the OB neurons in the anesthetized and awake rats under odor stimulation. We observed that the LFP oscillations of anesthetized and awake rats were modulated to varying degrees by odor stimulation. Our results showed that the δ, θ, *β*, and high-frequency power were enhanced, and the *γ* power decreased in the awake rats during odor stimulation. In addition, the δ power of anesthetized rats decreased during odor stimulation, θ and *β* power increased, and *γ* and high-frequency oscillations did not respond to odor stimulation. The decrease of *γ* power in awake animals was accompanied by the enhancement of *β* power, consistent with the findings of previous studies (Beshel et al., 2007; Fuentes et al., 2008). However, we did not observe this phenomenon in anesthetized animals. Previous studies have shown that *γ* oscillation is more prominent in awake animals than in anesthetized animals (Li et al., 2012). As a result, we observed more robust *γ* oscillation and *γ* responses to odor stimuli in awake animals. The slow oscillation of 1‒4 Hz exists in the OB of anesthetized and awake rats, also called respiratory rhythm. Recent studies have shown that this slow oscillation is affected by the respiratory input and by internal processing (Lockmann et al., 2016; Kocsis et al., 2018). Our study found that the δ rhythm is modulated differently by the odor stimuli during anesthesia and when awake, suggesting that this slow oscillation is also related to the brain state during sensory processing. In addition, our study found that the odor stimuli also modulated the high-frequency oscillations of awake animals. This rapid oscillation in the anesthetized animals has been reported numerous times in other studies. Based on the quantitative analysis of the power spectrum of high-frequency oscillations in the rats under two states, we found that HFO has more substantial power under anesthesia, which may be due to the effect of anesthesia. Previous studies have shown that the subanesthetic state significantly enhanced HFO in the OB (Hunt et al., 2019). The θ oscillation in the OB in this study distinctly modulated the HGO and HFO amplitude activity during the free movement, and odor presentation significantly modified these modulations. Changes in the activity of frequency bands and their interactions in the OB suggest distinct physiological processes subserving different functions. For instance, the θ-HG coupling in CA1 may play a role in memory encoding (Colgin and Moser, 2010), while θ-HFO has a possible role for inhibitory interneurons (Klausberger and Somogyi, 2008). Therefore, appropriate behavior tasks need to be designed in the future to explore further the specific physiological significance of oscillations in different frequency bands in the OB and how their interaction participates in olfactory processing.

## Data Availability

The original contributions presented in the study are included in the article/Supplementary Material; further inquiries can be directed to the corresponding authors.
