# Supplementary material for: Odor-induced modification of oscillations and related theta-higher gamma coupling in olfactory bulb neurons of awake and anesthetized rats
Source: Front Chem. 2022 Aug 1;10:865006. doi: 10.3389/fchem.2022.865006 (PMC9376862; doi:10.3389/fchem.2022.865006)
Supplement: Supplementary file 2 [file DataSheet1.docx]

**Odor Induced Modification of Oscillations and Related θ-Higher Gγ Coupling in Olfactory Bulb Neurons of Awake Rats**

Ping Zhu ^1,2^, Shuge Liu^1,2^, Yulan Tian^1,2^, Yating Chen^1,2^, Wei Chen^1,2^, Ping Wang^3^, Liping Du^1,2,3*^, Chunsheng Wu^1,2*^

1 (Institute of Medical Engineering, Department of Biophysics, School of Basic Medical Sciences, Health Science Center, Xi’an Jiaotong University, Xi’an 710061, China)

2 (Key Laboratory of Environment and Genes Related to Diseases (Xi'an Jiaotong University), Ministry of Education of China, 710061, Xi'an, China)

3 (Biosensor National Special Laboratory, Key Laboratory for Biomedical Engineering of Ministry of Education, Department of Biomedical Engineering, Zhejiang University, Hangzhou 310027, China)

* Correspondence: L. Du (duliping@xjtu.edu.cn) and C. Wu (e-mail: [wuchunsheng@xjtu.edu.cn](mailto:wuchunsheng@xjtu.edu.cn))

**Supplementary materials**


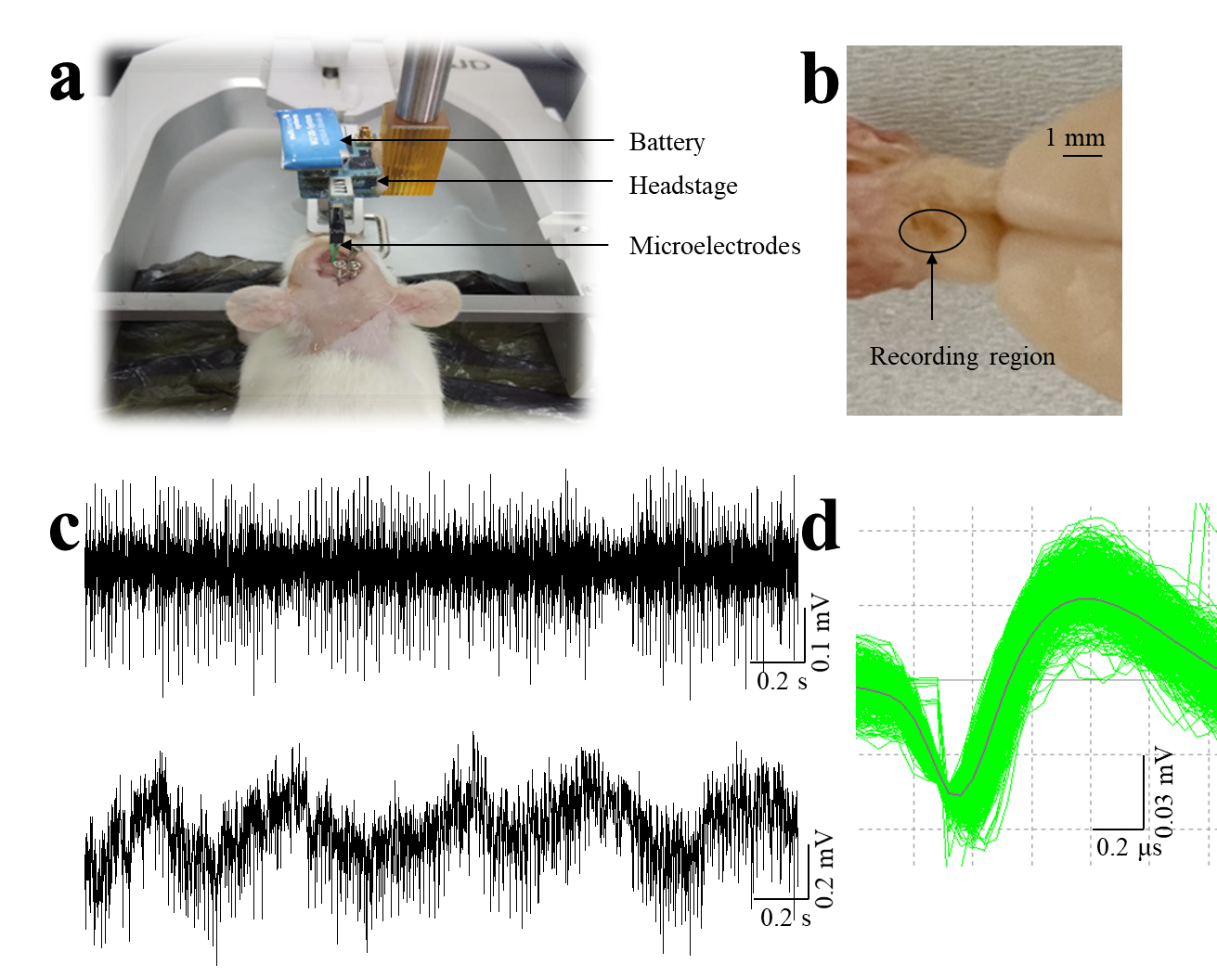


Fig. S1 Details on the recording’s setup. (a) Example of anesthetized rat with MEA headstage. (b) The olfactory bulb recording region. (c) Example of neuron firing activity (200 Hz high-pass filtering, upper) and raw signal (below) in olfactory bulb. (d) Spike waveforms sorted out from the neuron in (c).


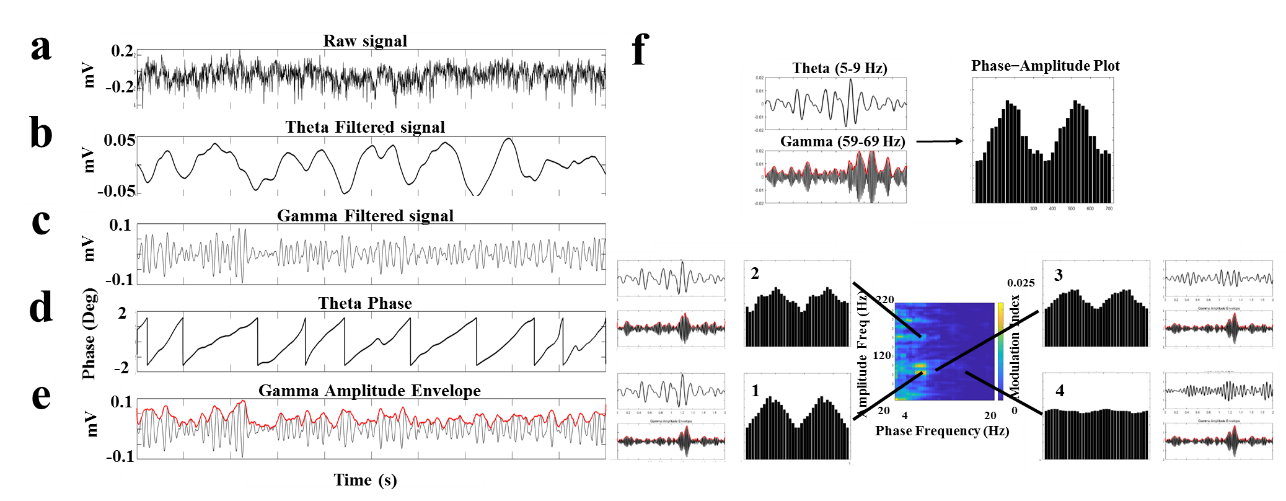


Fig.S2 Steps in the computation of cross-frequency coupling and modulation index (MI). The raw signal (a) is filtered to obtain theta (b) and gamma (c) frequency, respectively. Their phase (d) and amplitude envelope (e) time series are then calculated by using a Hilbert transform. A phase-amplitude distribution (f, left and middle) is obtained by computing the mean amplitude over each phase bin (2 cycles shown). The comodulogram (g) is obtained by calculating the MI of multiple band pairs and display in the results on the 2D pseudo-color map. The MI values of the four points in (g) are 0.023 (1), 0.015 (2), 0.0023 (3) and 0.00007 (4), respectively.

The original code

#CFC

Fs=25000;

wave = load('X.mat');

wave = wave.wave;

wave = wave';

y=wave(Tstart*Fs+1:Tend*Fs);

%M = [];

for ii = 1:1:18

WSPK = [(ii)*2,(ii+4)*2];

OmegacSPK =WSPK/Fs;

[numdSPK,dendSPK] = butter(2,OmegacSPK,'bandpass');

a=filter(numdSPK,dendSPK,y);

t=(0:length(a)-1)/Fs;

y1=hilbert(a);

r1=real(y1);

i1=imag(y1);

P=atan2(i1,r1);

for j = 18:1:230

WSPK = [j*2,(j+10)*2];

OmegacSPK =WSPK/Fs;

[numdSPK,dendSPK] = butter(2,OmegacSPK,'bandpass');

b=filter(numdSPK,dendSPK,y);

t=(0:length(b)-1)/Fs;

z=hilbert(b');

A=abs(z);

[MI,distKL]=modulationIndex(P,A,18);

N1 = ii*2;

n1 = int8(N1);

M(n1,j*2) = MI;

end

end

#modulationIndex

Code is online

Pierre Mégevand (2022). modulation_index (https://github.com/pierremegevand/modulation_index), GitHub. Retrieved April 19, 2022.

#Wavelet

[wt,f,coi] = cwt(x,'amor',Fs);

pcolor(t,f,abs(wt));shading interp

#FFT

wSPKL = 4;

wSPKH =12;

WSPK = [wSPKL*2,wSPKH*2];

OmegacSPK =WSPK/Fs;

[numdSPK,dendSPK] = butter(2,OmegacSPK,'bandpass');

a1=filter(numdSPK,dendSPK,wave);

t=[0:length(a1)-1]/Fs;

T=1/Fs;

L=length(a1);

t=(0:L-1)*T;

Y=fft(a1);

P2=abs(Y/L);

P1=P2(1:L/2+1);

P1(2:end-1) = 2*P1(2:end-1);

f = Fs*(0:(L/2))/L;

figure(1);

plot(f,P1,'k','linewidth',4);

xlim([0,20]);

title('Single-Sided Amplitude Spectrum of X(t)');

xlabel('f (Hz)');

ylabel('|P1(f)|');
